# Diagnostic Accuracy of DaTQUANT^®^ Versus BasGanV2™ for ^123^I-Ioflupane Brain SPECT: A Machine Learning-Based Differentiation of Parkinson’s Disease and Essential Tremor

**DOI:** 10.3390/biomedicines13102367

**Published:** 2025-09-27

**Authors:** Barbara Palumbo, Luca Filippi, Andrea Marongiu, Francesco Bianconi, Mario Luca Fravolini, Roberta Danieli, Viviana Frantellizzi, Giuseppe De Vincentis, Angela Spanu, Susanna Nuvoli

**Affiliations:** 1 Section of Nuclear Medicine and Health Physics, Department of Medicine and Surgery, Università degli Studi di Perugia, Piazza Lucio Severi 1, 06132 Perugia, Italy; 2 Nuclear Medicine Division, Azienda Ospedaliera di Perugia, S. Andrea delle Fratte, 06156 Perugia, Italy; 3 Department of Biomedicine and Prevention, University of Rome ‘Tor Vergata’, Via Montpellier 1, 00133 Rome, Italy; 4 Nuclear Medicine Research Unit, IRCCS San Raffaele Roma, Via della Pisana 235, 00163 Rome, Italy; roberta.danieli@uniroma5.it; 5 Unit of Nuclear Medicine, Department of Medicine, Surgery and Pharmacy, University of Sassari, Viale San Pietro 8, 07100 Sassari, Italy; amarongiu2@uniss.it (A.M.); aspanu@uniss.it (A.S.); smfnuvoli@uniss.it (S.N.); 6 Department of Engineering, Università degli Studi di Perugia, Via Goffredo Duranti 93, 06125 Perugia, Italy; francesco.bianconi@unipg.it (F.B.); mario.fravolini@unipg.it (M.L.F.); 7 Department of Human Sciences and Promotion of the Quality of Life, University San Raffaele, Via di Val Cannuta 247, 00166 Rome, Italy; 8 Department of Radiological Sciences, Oncology and Anatomical Pathology, Sapienza University of Rome, 00161 Rome, Italy; viviana.frantellizzi@uniroma1.it (V.F.); giuseppe.devincentis@uniroma1.it (G.D.V.)

**Keywords:** Parkinson’s disease, DaTQUANT^®^, BasGanV2™, dopamine transporters, SPECT, ^123^I-Ioflupane, machine learning, artificial intelligence, rehabilitation, movement disorders

## Abstract

**Background**: Differentiating Parkinson’s disease (PD) from essential tremor (ET) is often challenging, especially in early or atypical cases. Dopamine transporter (DAT) single-photon emission computed tomography (SPECT) with ^123^I-Ioflupane supports diagnosis, and semi-quantitative tools such as DaTQUANT^®^ and BasGanV2™ provide objective measures. This study compared their diagnostic performance when integrated with supervised machine learning. **Methods**: We retrospectively analysed ^123^I-Ioflupane SPECT scans from 169 patients (133 PD, 36 ET). Semi-quantitative analysis was performed using DaTQUANT^®^ v2.0 and BasGanV2™ v.2. Classification tree (ClT), k-nearest neighbour (k-NN), and support vector machine (SVM) models were trained and validated with stratified shuffle split (250 iterations). Diagnostic accuracy was compared between the two software packages. **Results**: All classifiers reliably distinguished PD from ET. DaTQUANT^®^ consistently achieved higher accuracy than BasGanV2™: 93.8%, 93.2%, and 94.5% for ClT, k-NN, and SVM, respectively, versus 90.9%, 91.7%, and 91.9% for BasGanV2™ (*p* < 0.001). Sensitivity and specificity were also consistently higher for DaTQUANT^®^ than BasGanV2. Class imbalance (PD > ET) was addressed using Synthetic Minority Over-sampling Technique (SMOTE). **Conclusions**: Machine learning analysis of ^123^I-Ioflupane SPECT enhances differentiation between PD and ET. DaTQUANT^®^ outperformed BasGanV2™, suggesting greater suitability for AI-driven decision support. These findings support the integration of semi-quantitative and AI-based approaches into clinical workflows and highlight the need for harmonised methodologies in movement disorder imaging.

## 1. Introduction

The differential diagnosis of movement disorders—such as Parkinson’s disease (PD) and parkinsonian syndromes versus essential tremor (ET)—relies primarily on clinical presentation. However, it is often challenging and therefore requires the integration of all available data to reach a more reliable diagnosis. In this context, dopamine transporter (DAT) single-photon emission computed tomography (SPECT) using ^123^I-Ioflupane has become a cornerstone in the evaluation of patients presenting with parkinsonian syndromes and tremor disorders [1,2,3]. This technique enables in vivo visualisation of presynaptic dopaminergic function, thereby facilitating the distinction between degenerative conditions such as PD and non-degenerative tremor disorders, notably ET. While expert visual interpretation remains the clinical standard, the subjective nature of qualitative assessment can introduce interobserver variability and diagnostic uncertainty, particularly in early-stage or atypical presentations [4].

To address these limitations, semi-quantitative analysis tools have been developed, providing objective measures of specific binding ratios (SBR) within anatomically defined regions of interest (ROI) [5]. Among the most widely used platforms are DaTQUANT^®^ (GE Healthcare) and BasGanV2™ (Associazione Italiana di Medicina Nucleare, AIMN) [6,7]. These systems automate processes such as image reorientation, ROI placement, and, in the case of BasGanV2™, partial volume effect correction. Although several studies have demonstrated strong correlations between the two approaches, methodological differences may bias uptake values and derived indices, potentially influencing clinical interpretation [7].

In parallel with advances in image acquisition and quantification, artificial intelligence (AI), and machine learning are reshaping medical imaging analysis [8,9]. AI-driven classifiers, including decision trees, support vector machines and neural networks, have shown the ability to process high-dimensional data, detect subtle patterns, and enhance diagnostic accuracy across a variety of radiological domains [10,11]. In neuroimaging, these methods are increasingly applied to identify disease-specific signatures, predict disease progression, and support clinical decision-making, thereby complementing traditional evaluation methods and contributing to personalised medicine [10,12,13]. Nevertheless, the use of AI-based tools in nuclear neuroimaging remains relatively underexplored, particularly in the context of movement disorders, where they could play a valuable role in both image interpretation and quantitative analysis [14,15].

The aim of our study was to investigate the diagnostic utility of two semi-quantitative SPECT tools—DaTQUANT^®^ and BasGanV2™—when integrated with supervised machine learning classifiers for the automated differentiation of PD and ET. By evaluating classifier performance metrics, we sought to clarify how differences in ROI methodology and partial volume correction influence AI-driven decision support in movement disorders. Ultimately, our findings are intended to inform best practices for the integration of semi-quantitative and AI-based techniques into routine clinical workflows.

## 2. Materials and Methods

A ^123^I-Ioflupane brain SPECT scan with semi-quantitative analysis, using the DaTQUANT^®^ and BasGanV2™ software packages [7], was performed in 169 retrospective patients (71 females, 98 males; mean age 69.2 ± 8.6 years, range 35.5–84.7 years) referred for the investigation of movement disorders characterised by tremor, bradykinesia and gait disturbances.

Of the studied cohort, 133 patients were clinically diagnosed as affected by PD [86 males and 47 females; age range 44–85 years; Hoehn and Yahr (HY) stage: 0.5–1.5; Unified Parkinson’s Disease Rating Scale (UPDRS) score: 6–38], and 36 patients were classified as affected by ET [12 males, 24 females; age range 37–82 years], as detailed in Table 1. The duration of symptoms at presentation ranged from 6 to 18 months, and all scans were performed prior to beginning any specific anti-Parkinsonian therapy.

All patients also underwent magnetic resonance imaging (MRI) to exclude cerebrovascular disease. A minimum clinical follow-up of six months confirmed the final diagnosis. For this retrospective study, all participants provided written informed consent authorising the anonymous use of their data for scientific purposes.

### 2.1. SPECT Protocol

^123^I-Ioflupane (185 MBq; DaTSCAN^®^, GE Healthcare, Medi-Physics Inc., Arlington Heights, IL, USA) was intravenously administered to each patient at least 30 min after oral administration of potassium perchlorate (400 mg) to block thyroid uptake of radiolabelled iodine. SPECT imaging was performed 3.5 h following radiopharmaceutical injection using a dual-head gamma camera (Millennium VG; General Electric Medical Systems, Milwaukee, WI, USA) equipped with low-energy high-resolution collimators.

The system was calibrated to the 159 keV photopeak with a ±10% energy window. The acquisition protocol included a 180° rotation for each detector head, a 128 × 128 frame matrix, a zoom factor of 1, a frame time of 30 s, and an angular step of 3°. Patients were positioned supine with the head immobilised in a dedicated holder to reduce movement artefacts. Image reconstruction was performed using filtered back-projection with a Butterworth filter (cut-off frequency 0.5; order 10). The resulting images were reformatted into transaxial, coronal, and sagittal planes aligned to the orbitomeatal line, with a slice thickness of 2.23 mm. All reconstructed brain SPECT images were independently reviewed by expert (with more than 15 years’ experience) nuclear medicine physicians (S.N., L.F. and B.P.), blinded to the patients’ clinical information. For qualitative analysis, scans were classified as follows: (1) normal, defined by homogeneous and symmetric tracer uptake in the caudate and putamen nuclei; (2) abnormal, defined by the presence of symmetric or asymmetric striatal uptake tracer reduction. All discrepancies were resolved by consensus, resulting in negligible inter-observer variability.

### 2.2. Image Processing

In the post processing phase the reconstructed images were analysed for semi-quantitative evaluation with both DaTQUANT^®^ (GE Healthcare) and the freely available BasGanV2™ software (https://aimn.it/associazione/gruppi-di-studio/, accessed on 29 August 2025).

DaTQUANT^®^ software reconstructed the images with the same algorithm and filter parameters applied to the normal database. The software automatically delineated bilateral volumes of interest (VOIs) within the striatum, caudate, putamen, anterior putamen, and posterior putamen. The occipital cortex was used as the reference background region. For each VOI, quantitative parameters comprised the striatal binding ratio (SBR; determined as the difference between mean counts in the region of interest and the background, normalised by the mean background counts), the percentage deviation from the age-adjusted mean of the normative database, the corresponding z-score, and the age-adjusted mean value. Age-matching was performed on a year-by-year basis, using a normative database composed of 118 healthy volunteers (73 men and 45 women, aged 31–84 years) who had neither a diagnosis of Parkinson’s disease nor a first-degree relative affected by the disease.

**Figure 1:**
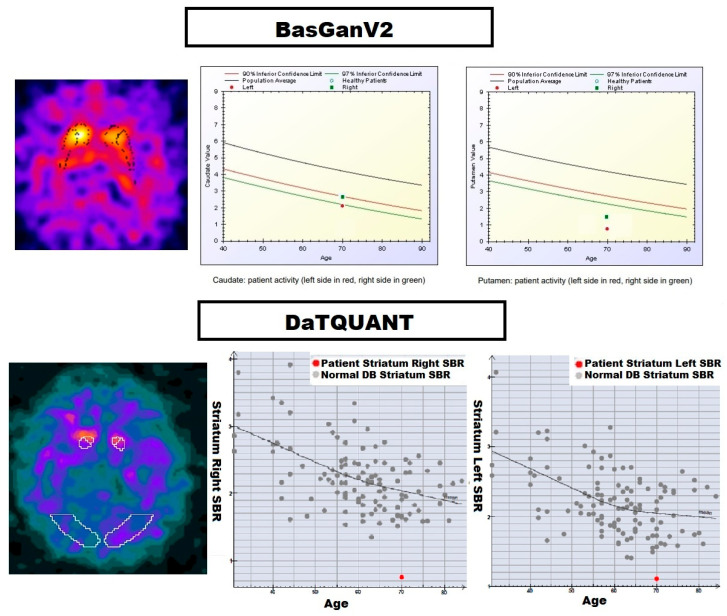
Example representation of the 3D striatal template positioning in the SPECT transaxial sections, along with the SBR for the right and left striatum for BasGanV2™ (upper row) and DaTQUANT^®^ (lower row). On the left side, the colour scale in the images represents different levels of activity/intensity. Red/yellow areas indicate regions with the highest levels of activity (or tracer uptake); pink/purple areas correspond to intermediate levels of activity: blue/dark areas represent regions with the lowest levels of activity. On the right of the lower row, in the graphs the central line represents the regression line fitted to the data points. It summarizes the average relationship between age (*x*-axis) and striatal SBR (*y*-axis).

BasGanV2™ employs a high-definition three-dimensional striatal template derived from the Talairach atlas, as previously described elsewhere [16]. The software enables automated three-dimensional segmentation of the caudate nucleus and putamen in both hemispheres. An optimisation algorithm refines the alignment of blurred templates to best correspond with the radioactive signal, while also defining an occipital region of interest (ROI) for background assessment. Correction for partial volume effects (PVE) is incorporated during the calculation of binding in the caudate nucleus, putamen, and occipital background. This correction relies on activity allocation within a Talairach–Tournoux atlas-based, three-compartment model of the basal ganglia. Finally, specific binding ratios (SBR) for the caudate nucleus and putamen in each hemisphere were derived by subtracting the background binding according to the following formula: [(binding in caudate nucleus or putamen—background binding)/background binding]. Figure 1 shows the 3-D striatal template positioning in the SPECT transaxial sections for BasGanV2™ and DaTQUANT^®^, respectively.

### 2.3. Statistical Analysis

Statistically significant differences in the radiopharmaceutical uptake values from the different areas between PD and ET were assessed through non-parametric, two-sided Mann–Whitney U test. *p*-values < 0.05 were considered significant after Bonferroni correction for multiple tests.

### 2.4. Machine Learning Analysis

A machine learning analysis was carried out to determine the ability of radiopharmaceutical uptake to discriminate PD vs. ET. For this task we tested three classification models: classification tree (ClT), k-nearest neighbour (k-NN), and support vector machine (SVM).

#### 2.4.1. Hyperparameter Tuning

Hyperparameter tuning was carried out by 5-fold cross validation on the training set and an exhaustive grid-search over specified parameter values as detailed below.

- ClT
  - Measure of the quality of a split = {‘gini’, ‘entropy’}
  - Depth of the tree = {2, 3, 4}
- k-NN
  - Number of neighbours = {1, 2, 3, 4}
- SVM
  - Regularisation parameter *C* = {0.1, 1, 10, 100, 1000}
  - Kernel = {‘linear’, ‘rbf’}
  - Kernel coefficient for ‘rbf’ = {1, 0.1, 0.01, 0.001, 0.0001}

For all the other parameters we used the default settings available in scikit-learn.

#### 2.4.2. Performance Estimation

A stratified shuffle split with a 70% training ratio and 250 random subdivisions into training and test sets (splits) served as the basis for accuracy estimation. In each split 70% of the samples were randomly sampled from each of the PD and ET groups and assigned to the training set, the remaining 30% to the test set for performance estimation. Accuracy, sensitivity, and specificity were recorded as the figures of merit, with the results averaged over the 250 splits.

Following the approach proposed in [17], preliminary Synthetic Minority Over-sampling Technique (SMOTE) was carried out to counteract class imbalance and raise the ratio of the minority class (ET) of the total from the original 21% to 35%.

All the analyses were carried out in Python with functions from NumPy, pandas, scikit-learn, and SciPy. Data visualisation was based on Python and Seaborn [18,19].

## 3. Results

The mean radiopharmaceutical uptake values obtained by ^123^I-Ioflupane brain SPECT scans using the two different software packages for semi-quantitative analysis (DaTQUANT^®^ and BasGanV2™) in both PD and ET patients are reported in Table 2.

All the automatic classifiers, ClT, k-NN, and SVM, were able to accurately classify the two groups of patients by extracting features from data derived from both DaTQUANT^®^ and BasGanV2™ software (Table 3).

DaTQUANT^®^ was more accurate than BasGanV2, showing a significantly higher accuracy (*p* < 0.001) when discriminating between PD and ET (93.8%, 93.2%, 94.5%), as compared to BasGanV2™, which had an accuracy of 90.9%, 91.7%, and 91.9%. Sensitivity and specificity were also consistently higher for DaTQUANT^®^ than BasGanV2 (Table 3).

**Figure 2:**
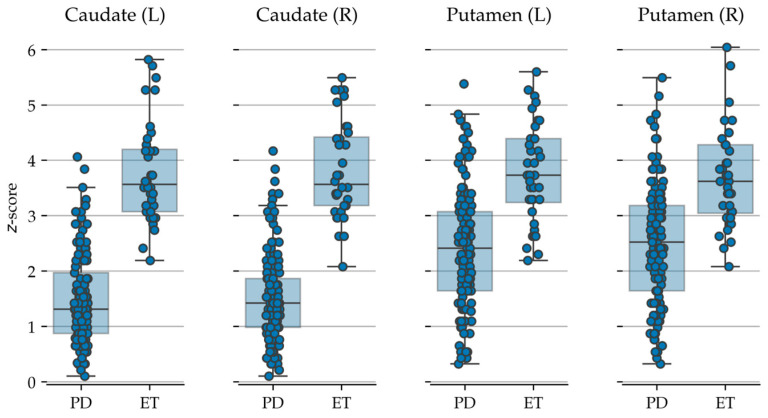
Box-plots/strip-plots showing the data generated with BasGanV2™. Each dot represents one subject. Key to abbreviations: PD = Parkinson’s disease, ET = essential tremor, L = left, R = right.

**Figure 3:**
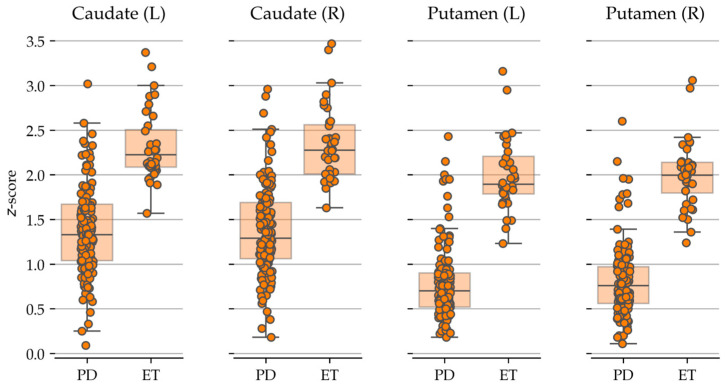
Box-plots/strip-plots showing the data generated with DaTQUANT^®^. Each dot represents one subject. Key to abbreviations: PD = Parkinson’s disease, ET = essential tremor, L = left, R = right.

Figure 2 and Figure 3 represent the semi-quantitative data obtained with BasGanV2™ and DaTQUANT^®^, respectively.

## 4. Discussion

Although previously published papers have discussed the role of semi-quantitative analysis of ^123^I-Ioflupane brain SPECT data in movement disorders [20,21,22,23], our paper is the first to compare two different semi-quantitative analysis tools, namely DaTQUANT^®^ and BasGanV2™, by using different AI classifiers to evaluate their diagnostic performance in discriminating PD vs. ET.

The same two software packages were compared with each other and also with a reading by experts in a previous paper by Morbelli and co-workers [7] investigating data from 151 patients with suspected PD undergoing brain SPECT with ^123^I-Ioflupane. The authors concluded that both software packages performed well and yielded comparable semi-quantitative results for DAT SPECT, although each has distinct strengths and limitations that users should understand.

In this paper we studied patients with PD and ET by using ^123^I-Ioflupane brain SPECT with both of the mentioned semi-quantitative software packages to critically compare their ability to contribute to the differential diagnosis, examining data with automatic classifiers. In other papers from our group [24,25], we applied automatic classification techniques to detect the capability of brain SPECT data when analysed with semi-quantification tools to diagnose PD patients, but the novelty of the present work is to compare the two most-used software tools in common practice with AI classifiers, as it is widely known that AI-based automated classifiers can provide a more reliable and refined contribution with respect to conventional statistical analysis [10,26,27]. The use of machine learning techniques in the available literature on nuclear medicine modalities to diagnose neurological diseases (i.e., movement disorders) is constantly increasing, as they can play a pivotal role, paving the way towards personalised medicine. Machine learning techniques can, from selected available data, learn the response characteristics of a system and construct a mathematical model capable of classifying previously unseen cases [10]. This is particularly relevant to the diagnostic process, as it aids the interpretation of new data and facilitates decision-making. However, the complexity of such models requires a multidisciplinary approach, with engineers and physicists collaborating closely with clinicians.

Furthermore, in our series of patients, although DaTQUANT^®^ had higher accuracy than BasGanV2™ with all the ML classifiers employed, both of the tools were useful in discriminating between PD and ET, showing very good results. This means that, in terms of clinical practice, both tools are valuable and helpful in supporting nuclear medicine physicians to obtain a correct diagnosis.

As a limitation of the study, we have to remark that the number of PD subjects enrolled was higher than that of ET patients, which is, however, consistent with the higher prevalence of PD vs. ET in the general population.

Beyond metric reporting, interpretability of the machine learning decision process is essential before clinical translation. A further step forward in the field would be to add feature-importance analyses (for example, permutation importance or SHAP values) to characterise which SBR regions or derived indices drive classifier decisions, and to assess whether these correspond to established pathophysiological markers (e.g., posterior putamen involvement in early PD) [28,29]. External validation on an independent cohort — ideally acquired with different cameras and reconstruction settings—is also necessary to assess generalisability, given the documented proportional bias between BasGanV2™ and DaTQUANT^®^ [30].

Importantly, methodological discrepancies between DaTQUANT^®^ and BasGanV2™—including differences in VOI definition (fixed versus atlas- or segmentation-based approaches), reference region selection, and age-matching procedures, the application or omission of partial-volume correction, and the underlying segmentation and quantification algorithms—can introduce both systematic and proportional biases in SBR estimates. These biases are most critical when SBR values fall close to established diagnostic thresholds: even small absolute differences between software packages may shift categorical classification, thereby reducing sensitivity for detecting very early Parkinson’s disease or generating discordant results in atypical tremor presentations [31,32]. Further variability may arise from acquisition- and reconstruction-related factors (e.g., smoothing and iterative reconstruction parameters) and from the distinct normative databases used to compute age-adjusted z-scores. Clinically, this implies that semi-quantitative SBRs should be interpreted cautiously and always in conjunction with the broader clinical picture; in cases of diagnostic uncertainty, longitudinal imaging to assess change over time, corroboration with clinical biomarkers (such as dopaminergic treatment response or validated rating scales), and, where available, complementary imaging modalities can provide additional reassurance [33]. Finally, integrating clinical variables (age, symptom lateralisation, UPDRS motor score) may further improve model robustness and should be explored in subsequent prospective studies.

To strengthen the evidence base, a prospective, multicentre validation study enrolling adequately powered and balanced PD and ET cohorts is recommended; such a study should define prespecified diagnostic endpoints, adopt harmonised imaging protocols with blinded central reading, and report standardised performance metrics to enable robust assessment of diagnostic accuracy and model calibration across scanners and settings.

## 5. Conclusions

In conclusion, machine learning analysis of ^123^I-Ioflupane SPECT derived from commonly used semi-quantitative software packages improved differentiation between Parkinson’s disease and essential tremor. Notably, DaTQUANT^®^ marginally outperformed BasGanV2™, suggesting it may be better suited for integration into AI-driven decision-support tools for movement-disorder imaging.

## Figures and Tables

**Table 1:** Patient Cohort Characteristics.

Group	Number of Patients	Gender (M/F)	Age Range (Years)	HY Stage	UPDRS Score
Parkinson’s Disease (PD)	133	86/47	44–85	0.5–1.5	6–38
Essential Tremor (ET)	36	12/24	37–82	N/A	N/A

PD: Parkinson’s Disease; ET: Essential Tremor; M: Male; F: Female; HY: Hoehn and Yahr stage; UPDRS: Unified Parkinson’s Disease Rating Scale; N/A: not available.

**Table 2:** Uptake values of brain SPECT with ^123^I-Ioflupane in Parkinson’s disease (PD) and Essential Tremor (ET) patients in Putamina (Right and Left) and in Caudate Nuclei (Right and left), expressed as mean ± standard deviation.

Area	Semi-Quantitative Analysis Tool	PD	ET	*p*-Value	*p*-Value (Corrected)
**Caudate (L)**	**DaTQUANT** ^®^	1.37 ± 0.49	2.32 ± 0.39	<0.001	<0.001
**Caudate (R)**		1.38 ± 0.50	2.33 ± 0.43	<0.001	<0.001
**Putamen (L)**		0.78 ± 0.41	2.00 ± 0.40	<0.001	<0.001
**Putamen (R)**		0.81 ± 0.40	2.00 ± 0.38	<0.001	<0.001
**Caudate (L)**	**BasGanV2™**	1.50 ± 0.81	3.77 ± 0.92	<0.001	<0.001
**Caudate (R)**		1.52 ± 0.81	3.82 ± 0.88	<0.001	<0.001
**Putamen (L)**		2.44 ± 1.07	3.76 ± 0.90	<0.001	<0.001
**Putamen (R)**		2.49 ± 1.07	3.69 ± 0.90	<0.001	<0.001

**Table 3:** Classification accuracy, sensitivity, and specificity of the three classifiers (ClT, K-NN, and SVM) to discriminate patients with PD and ET, showing that DaTQUANT had a significantly higher accuracy with respect to BasGan V2 (*p* < 0.001). Key to abbreviations: Acc = accuracy, Sn = sensitivity, Sp = specificity. Values are in %.

	ClT			K-NN			SVM		
	Acc	Sn	Sp	Acc	Sn	Sp	Acc	Sn	Sp
DaTQUANT^®^	93.8	92.7	96.0	93.2	93.2	93.1	94.5	92.8	97.5
BasGanV2™	90.9	90.2	92.0	91.7	91.0	92.9	91.9	91.2	93.1
*p*-Value (Acc)	<0.001			<0.001			<0.001		

## Data Availability

The original contributions presented in this study are included in the article. Underlying data will be made available upon reasonable request to the corresponding author.
